# Discrete, high-latitude foraging areas are important to energy budgets and population dynamics of migratory leatherback turtles

**DOI:** 10.1038/s41598-018-29106-1

**Published:** 2018-07-20

**Authors:** Bryan P. Wallace, Michael Zolkewitz, Michael C. James

**Affiliations:** 1 Conservation Science Partners, Inc., 5 Old Town Square, Suite 205, Fort Collins, CO 80524 USA; 2 Nicholas School of the Environment, Duke University, Beaufort, NC USA; 3 Ecological Research Solutions, New Hope, PA USA; 4 Population Ecology Division, Fisheries and Oceans Canada, Dartmouth, Nova Scotia B2Y 4A2 Canada

## Abstract

Many broadly distributed migratory species exhibit fidelity to fine-scale areas that support vital life history requirements (e.g., resource acquisition, reproduction). Thus, such areas are critical for population dynamics and are of high conservation priority. Leatherback sea turtles are among the world’s most widely distributed species, and their breeding and feeding areas are typically separated by thousands of kilometres. In this study, we analysed turtle-borne video data on daytime feeding rates and energy acquisition in Nova Scotia, Canada, to quantify the importance of this discrete, seasonal foraging area for leatherback energy requirements. Based on daytime foraging only, we estimate that a single foraging season in Nova Scotia could support 59% of a non-breeding leatherback’s annual energy budget, and 29% of energetic requirements for a female on a typical 2-year reproductive cycle. However, maximum energy intake rates for leatherbacks are nearly four times lower than those of mammals and birds due the low energy content of leatherbacks’ gelatinous zooplankton prey. These results illustrate that high quality, local-scale foraging areas such as Nova Scotia are critically important to the stability and future growth of the leatherback population in the Northwest Atlantic Ocean. Thus, as with other migratory species, efforts to reduce threats and maintain habitat quality in such areas should be high conservation priorities.

## Introduction

Migratory species present unique challenges to management due to their broad distributions; utilization of multiple, distinct habitat types during their life cycles; and exposure to diverse threats of varying magnitude across time and space^1–5^. For marine species, efforts to assess conservation status and threats often focus on their broad-scale, international distributions, and on identification of overlaps with anthropogenic impacts, especially fishing activity^6–10^. This approach attempts to match species’ distributions and habitat use to management-relevant scales, and it describes areas important for conservation based on patterns of high use inferred from remote sensing^11^, high risk due to presence of threats and corresponding evidence of turtle-threat interaction^10^, or a combination of both^12,13^. Although this conceptual approach can highlight areas for conservation efforts within ocean basin-wide distributions, these areas are rarely—if ever—static in time and space, a limitation which has prompted more dynamic approaches to management^14^. Furthermore, important areas for migratory marine species often overlap with jurisdictions of multiple agencies, organizations, governments, and inter-governmental bodies with non-overlapping missions, a situation that typically prevents harmonized management schemes^4,7^.

Migratory marine species often exhibit remarkably fine-scale preferences for and fidelity to particular areas within their broad geographic distributions. These species have evolved suites of physiological and biological traits that allow them to detect and exploit areas that are critical to acquisition of resources in fulfilment of their life history requirements, namely reproduction^1,3,5^. Because these critical habitats have a disproportionate influence on population dynamics relative to their small spatial scales, identification of such areas provides conservation targets that are not only more logistically feasible to address, but that might also result in higher return on investments to reduce threats and to protect or enhance available habitats^3,15^.

Leatherback turtles (*Dermochelys coriacea*) are among the most widely distributed extant animal species, with breeding and nesting areas throughout the tropics and foraging areas encompassing boreal latitudes^16,17^. Leatherbacks routinely migrate thousands of kilometres between nesting beaches and foraging areas^11,16–19^, an energetically demanding feat fuelled by a highly-specialised diet of gelatinous zooplankton (i.e., jellyfish, salps, etc.)^16,17^. Several leatherback subpopulations are considered threatened with extinction^20^ due to a combination of population characteristics that make them susceptible to perturbations and anthropogenic threats, particularly incidental capture in fishing gear (i.e. bycatch), and human consumption of eggs and meat^7^. When threats and turtles are concentrated simultaneously in a particular area, population impacts can be disproportionately high, as illustrated by bycatch in small-scale fisheries^21,22^ and egg harvest by humans in various regions^23,24^. Even in regions where leatherback numbers may be stable currently, continuity of conservation efforts is necessary to ensure that those populations do not eventually decline in response to persistent threats^25^. Therefore, successful management of marine migratory species like leatherbacks depends on identifying and protecting critical habitats in order to safeguard future population health.

In the Northwest Atlantic Ocean (NWA), several leatherback foraging areas have been identified based on analyses of movements^12^ and direct observation^19,26^. In particular, long-term monitoring using in-water capture and satellite telemetry has documented relatively high numbers of leatherbacks in Canadian waters off Nova Scotia, including the southern Gulf of St. Lawrence, during July-October each year^18,27,28^. Leatherbacks feed almost exclusively on large scyphozoan jellyfish (e.g., *Cyanea capillata*, while in Canadian waters^16,29–31^, and apparently time their arrival to and departure from this foraging area to coincide with conditions that favour high abundance of these prey^32^. Tag returns, genetic analyses, and satellite telemetry have revealed that these annual cohorts of foraging leatherbacks comprise breeding and non-breeding adults as well as subadults from distinct breeding stocks from several sites throughout the Wider Caribbean^18,27,32–34^. However, though leatherbacks regularly appear in Canadian continental shelf waters, the actual importance of this area for supporting energetic requirements of reproduction, growth, and migrations for this subpopulation has not been quantified. Understanding of how migration affects other life history activities and how these interactions could influence conservation and management of migratory species remain understudied^35^.

**Figure 1:**
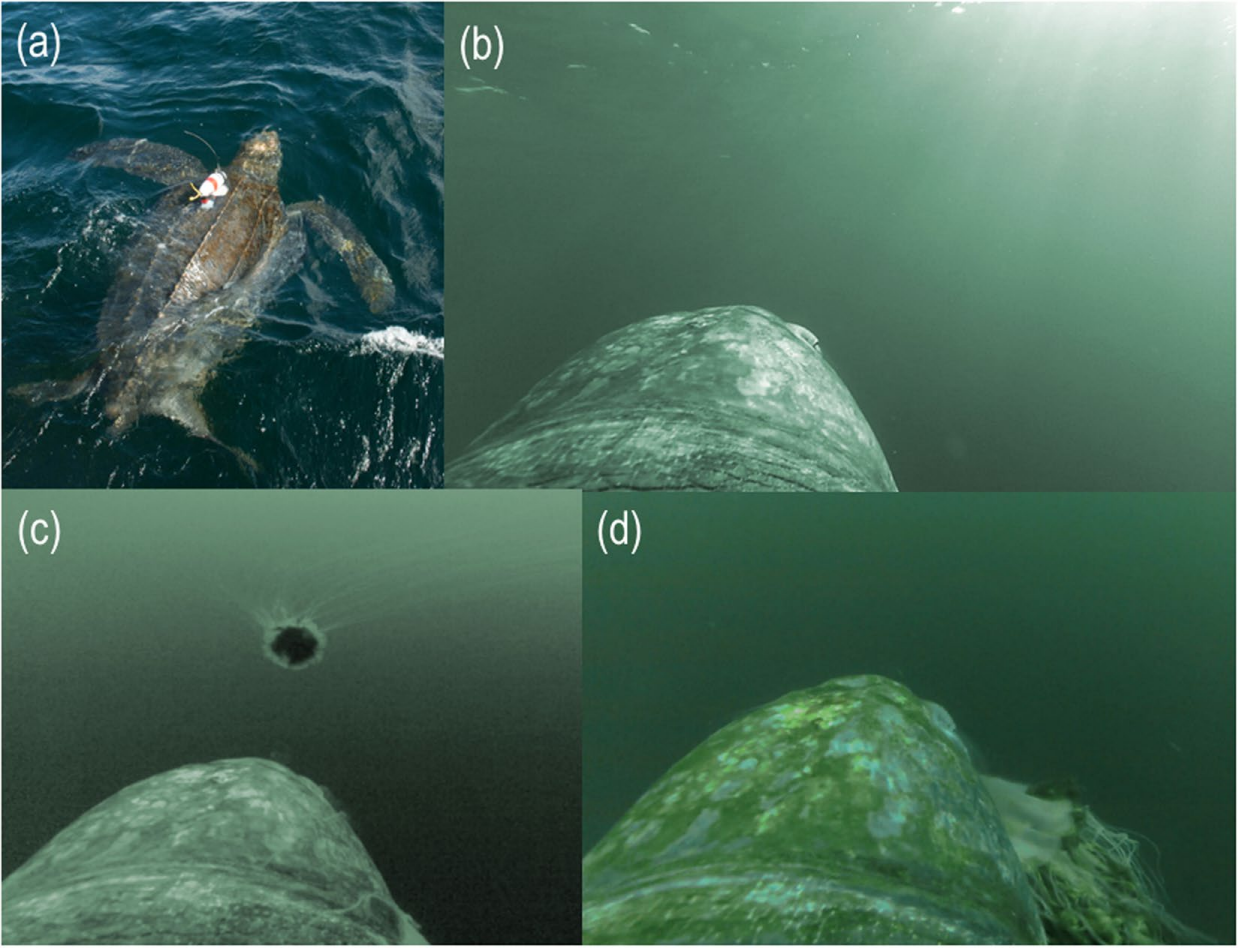
Turtle-borne video cameras with time-depth recorders (shown on a leatherback, [**a**]) obtained paired fine-scale video and dive data from leatherback turtles searching (**b**), locating (**c**), and capturing jellyfish prey (**d**) in Nova Scotia, Canada.

In this study, we obtained fine-scale data on leatherback daytime feeding rates and energy acquisition across multiple seasons to quantify the importance of a discrete, seasonal feeding area in Atlantic Canada to bioenergetics of NWA leatherbacks. We re-analysed foraging behavioural video footage^30,31^ (Fig. 1) and included new data from additional animals to derive estimates of energy budgets for adult males and females—in breeding and non-breeding years—as well as subadults. We then combined estimates of daytime energy intake with estimates of energy costs related to foraging activities and thermoregulation in cold Nova Scotian waters to quantify the relative energetic importance of foraging in Canada to the overall leatherback energy budget. This bioenergetics approach provides a robust, quantitative illustration of the disproportionately large influence of fine-scale habitats to the ocean basin-scale life history of a marine migratory species. Our results demonstrate the importance of maintaining the integrity of these habitats to ensure future population sustainability.

## Results

### Leatherbacks prey sizes, energy content, and intake

Based on direct observations of leatherback feeding behaviour during daytime hours only, leatherbacks captured between 8 and 70 jellyfish per recorded foraging interval (2.2 hr ± 0.9 hr) at an average rate of 16 jellyfish hr^−1^ (Table 1). Average prey size was 27.7 cm overall, and ranged between average minimum and maximum sizes of 17.0 cm and 40.4 cm, respectively (Table 1). We found no significant relationship between number of prey captured and average prey size (p > 0.05).

**Table 1:** Recorded foraging interval durations, number and sizes of prey captured, and estimated prey mass consumed by leatherback turtles foraging off Nova Scotia, Canada. Prey sizes and wet mass per prey item are presented as intra-individual averages for all prey sizes estimated for each turtle.

Turtle ID	Date	Time (Atlantic Daylight Time)	Sex	Recorded foraging interval duration (hr)	Jellyfish captured	Head always in view	Measuring efficiency	Jellyfish bell diameter (cm)			Average wet mass per jellyfish (kg)	Total mass consumed (kg)
								Average	Max	Min		
1	12 Aug 2008	10:37	F	0.92	32	Yes	50%	22.0	31.6	15.5	0.57	18.2
2	13 Aug 2008	13:02	U	1.71	48	No	81%	22.7	42.5	13.8	0.64	30.7
3	14 Aug 2008	11:27	F	2.94	58	Yes	71%	28.9	33.0	13.7	1.14	66.4
4	18 Aug 2008	14:02	F	1.61	40	No	38%	18.9	26.0	11.8	0.31	12.4
5	3 Sept 2008	10:33	M	1.16	27	No	81%	19.8	30.0	13.9	0.39	10.5
6	29 Aug 2009	14:11	F	1.40	19	No	74%	27.3	35.4	18.5	1.01	19.1
7	8 Sept 2009	12:04	M	1.88	36	No	15%	38.6	47.3	28.4	1.95	70.3
8	12 Sept 2009	10:49	F	0.93	16	Yes	100%	28.3	41.9	15.7	1.10	17.5
9	11 Aug 2010	11:51	M	2.61	70	No	46%	24.0	34.1	16.9	0.72	50.7
10	14 Aug 2010	11:47	F	2.23	8	No	63%	19.0	23.1	13.8	0.32	2.6
11	15 Aug 2010	17:41	F	2.20	35	No	92%	25.0	43.8	13.8	0.82	28.8
12	16 Aug 2010	11:45	U	3.31	34	No	82%	22.4	36.7	14.7	0.61	20.6
13	24 Aug 2010	12:29	M	3.40	32	Yes	94%	29.0	53.5	15.6	1.15	36.9
14	29 Aug 2010	10:12	U	3.51	47	Yes	96%	35.5	57.5	19.2	1.69	79.6
15	3 Sept 2010	13:25	M	1.86	22	Yes	91%	28.7	46.9	21.3	1.13	24.8
16	24 Aug 2011	10:06	F	2.84	23	Yes	83%	40.1	60.2	27.1	2.07	47.7
17	27 Aug 2011	13:14	U	3.55	59	Yes	93%	29.0	48.8	17.4	1.11	65.5
18	31 Aug 2011	09:29	U	1.27	13	No	62%	25.1	36.7	14.3	0.83	10.8
19	2 Sept 2011	12:28	U	2.59	30	Yes	90%	24.8	34.1	12.4	0.81	24.2
20	27 Aug 2013	15:10	U	1.87	14	Yes	57%	35.5	41.2	22.5	1.69	23.7
	**MEAN**			**2**.**19**	**33**.**2**		**73%**	**27**.**2**			**1**.**00**	**33**.**1**
	**SD**			**0**.**87**	**16**.**7**		**23%**	**6**.**2**			**0**.**52**	**22**.**6**

Measuring efficiency refers to the proportion of jellyfish detected that could also be measured; for the remainder, the average jellyfish size associated with each turtle was used to calculate jellyfish wet mass. Prey mass was estimated using prey size-mass formulas from ref.^40^.

Among turtles, average prey mass was 1.0 ± 0.5 kg and ranged between 0.6 and 2.1 kg. For entire observed foraging intervals, leatherbacks consumed between 10.8 kg to 66.4 kg, and averaged 33.1 kg (Table 1). Assuming daytime-only foraging and 14 hr day lengths^31,36,37^ (see Methods), average hourly and daily biomass consumption rates were 15.0 ± 7.8 kg hr^−1^ and 209.4 ± 109 kg d^−1^, or 228 ± 112 jellyfish d^−1^, respectively, with individual estimates ranging between 50 and nearly 500 jellyfish d^−1^ (Table 2).

**Table 2:** Estimates of total mass and energy consumed, prey biomass and energy consumption rates, and total prey items consumed daily by leatherback turtles foraging off Nova Scotia, Canada. Prey biomass and energy consumption values are presented as intra-individual averages for all prey items measured for each turtle.

Turtle ID	Jellyfish Captured	total mass consumed (kg)	total energy consumed (kJ)	Biomass consumption rate (kg/hr)	Biomass consumption rate (kg/day)	Energy Consumption/Hr (kJ)	Energy Consumption/day (kJ)	Jellies consumed per day
1	32	18.2	3,670	19.7	275	3,189	44,647	484
2	49	30.7	6,209	18.0	252	3,105	43,466	402
3	58	66.4	13,407	22.6	316	4,152	58,130	276
4	40	12.4	2,501	7.7	107	1,201	16,814	347
5	27	10.5	2,128	9.1	127	1,497	20,957	326
6	19	19.1	3,864	13.7	191	2,421	33,890	190
7	36	70.3	14,209	37.3	523	7,478	104,688	267
8	16	17.5	3,536	18.7	262	3,422	47,904	240
9	70	50.7	10,233	19.4	272	3,263	45,688	376
10	8	2.6	518	1.2	16	197	2,758	50
11	35	28.8	5,811	13.1	183	2,346	32,839	223
12	34	20.6	4,162	6.2	87	1,059	14,825	144
13	32	36.9	7,448	10.8	152	2,034	28,483	132
14	47	79.6	16,072	22.7	317	4,534	63,475	187
15	22	24.8	5,019	13.3	187	2,458	34,408	165
16	23	47.7	9,638	16.8	235	3,493	48,896	113
17	59	65.5	13,226	18.5	259	3,391	47,470	233
18	13	10.8	2,178	8.5	119	1,506	21,082	143
19	30	24.2	4,885	9.3	131	1,630	22,821	162
20	14	23.7	4,790	12.7	178	2,476	34,665	105
**MEAN**	**33**.**2**	**33**.**1**	**6**,**675**	**15**.**0**	**209**	**2**,**743**	**38**,**395**	**228**
**SD**	**16**.**7**	**22**.**6**	**4**,**560**	**7**.**8**	**109**	**1**,**566**	**21**,**922**	**112**

Energy content was calculated using prey size-energy content formulas from ref.^40^. Foraging was assumed to occur only during daytime hours (i.e., 14 hours per day) (refs^36,37^; see Methods for details).

Total estimated energy intake during observed daytime foraging intervals averaged 6,700 kJ (±4,600 kJ), which significantly increased with both number (r^2^ = 0.08, p < 0.05) and average size (r^2^ = 0.5, p < 0.01) of jellyfish captured. We estimated that turtles consumed approximately 2,700 ± 1,500 kJ hr^−1^ and 38,000 ± 22,000 kJ d^−1^ (Table 2). Estimated daily energy intake increased with daily number of prey consumed (r^2^ = 0.08; p = 0.05), but was more significantly related to daily prey biomass consumption as a function of turtle body mass (r^2^ = 0.98, p < 0.0001). We did not observe feeding rates during nighttime hours, and thus could not quantify energy intake rates beyond daytime hours for which we had video data (See Methods for further discussion).

### Heat loss and metabolic rates

Leatherbacks equipped with video-data recorders experienced average water temperatures (T_w_) ranging between 13 °C and nearly 20 °C (mean 17.2 ± 1.7 °C), and thermal gradients between body temperatures (T_b_) and T_w_ (T_g_) of between 6.8 and 13 °C (assuming mean T_b_ = 26.4 °C; ref.^37^) (Table 3). Average mass-specific metabolic rate required to maintain estimated T_g_ and meet demands of heat loss (*q*_T_) during daytime hours was 0.66 ± 0.16 W kg^−1^, and decreased approximately 20% during night-time hours (Table 3).

**Table 3:** Thermal conditions and physiological responses of leatherback turtles in Nova, Scotia, Canada. Mean water temperatures (Tw) experienced by leatherback turtles in Nova Scotia, Canada; estimated thermal gradients between internal body temperatures (Tb; estimated as 26.4 °C^37^) and Tw; total heat loss (Q_T_) via heat exchange across shell and flippers; as well as heat required to warm ingested prey (daytime feeding only); and the estimated metabolic rates required to meet total heat loss during the day (with prey consumption) and during the night (no prey consumption, hence no heat lost to prey ingestion).

Turtle ID	Mean water temperature (°C)	Thermal gradient (T_b_−T_w_; °C)	Total heat loss (*q*_T_; W)	Required metabolic rate (W kg^−1^), daytime (w/prey consumption)	Required metabolic rate (W kg^−1^), nighttime (no prey consumption)
1	14.2	12.2	407.1	0.92	0.70
2	16.3	10.1	324.1	0.76	0.58
3	15.3	11.1	377.2	0.89	0.64
4	16.7	9.7	269.2	0.63	0.56
5	16.9	9.5	266.9	0.64	0.55
6	17.5	8.9	243.6	0.69	0.54
7	17.2	9.2	369.6	0.87	0.53
8	16.7	9.7	334.8	0.70	0.54
9	17.5	8.9	311.8	0.64	0.49
10	18.6	7.8	195.2	0.46	0.45
11	13.4	13.0	367.3	0.96	0.77
12	16.2	10.2	276.9	0.65	0.59
13	18.3	8.1	265.6	0.51	0.44
14	17.1	9.3	316.4	0.74	0.54
15	19.3	7.1	213.9	0.50	0.41
16	16.3	10.1	344.1	0.64	0.54
17	19.4	7.0	221.1	0.52	0.40
18	18.8	7.6	245.3	0.58	0.44
19	19.6	6.8	191.1	0.45	0.39
20	18.7	7.7	251.0	0.47	0.41
**MEAN**	**17**.**2**	**9**.**2**	**289**.**6**	**0**.**66**	**0**.**53**
**SD**	**1**.**7**	**1**.**7**	**63**.**9**	**0**.**16**	**0**.**10**

### Bioenergetics quantification

**Figure 2:**
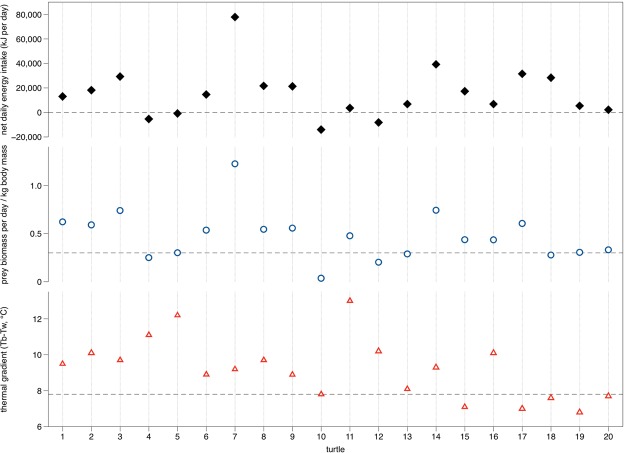
Net energy intake (kJ d^−1^) (top panel) for leatherback turtles feeding in Nova Scotia, Canada, increased with prey biomass intake rates (kg prey per kg turtle body mass d^−1^; middle panel) and maintenance of increased thermal gradients (difference between body temperatures [T_b_] and water temperatures [T_w_]; bottom panel). Negative net energy intake values (zero denoted by dotted line in top panel) were related to biomass intake rates ≤0.3 turtle body mass (dotted line in middle panel) and thermal gradients ≥7.8 °C (dotted line in bottom panel).

The difference between energy costs (e.g., swimming activity, thermoregulation) and energy acquisition through prey—i.e., the net energy intake—varied according to both the biomass intake rates (relative to body mass), as well as size of the thermal gradient between leatherback body temperatures and ambient temperatures (Fig. 2). Some turtles actually had negative net energy intake, which was mostly due to low biomass intake rates (≤0.3 kg prey per kg body mass), but also to maintenance of large thermal gradients (≥7.8 °C) (Fig. 2). Turtles achieved high net energy intake rates despite high costs of thermoregulation (i.e., maintenance of high thermal gradients) with high biomass intake rates. Likewise, net positive energy intake rates were possible even at relatively low biomass intake rates as long as thermal gradients (i.e., costs of thermoregulation) were low.

**Figure 3:**
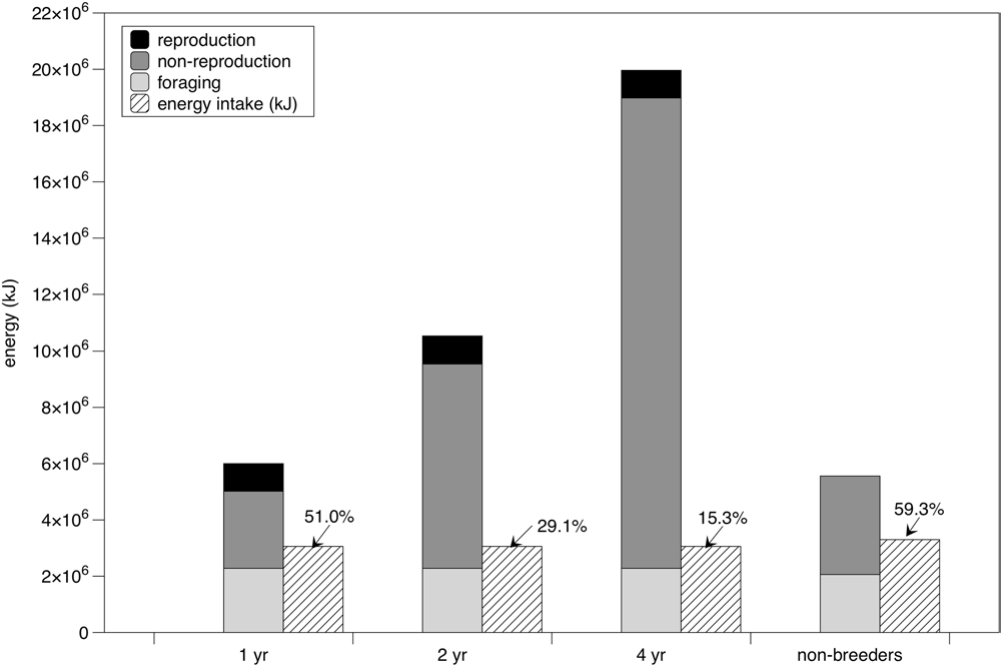
Energy budget estimates for leatherback turtles and the proportion of energy requirements that leatherbacks can acquire during a 90 d foraging period in Atlantic Canada (striped bars and percent values). Energy budgets calculated for breeding females with 1 yr, 2 yr, and 4 yr remigration intervals, and for non-breeding turtles (subadults, males, and females in a non-breeding year). See Methods for description of components of energy budget.

Estimated costs of foraging for 90d in continental shelf waters off Nova Scotia were similar among adult females (2.13 × 10^6^ kJ), subadults, and males (2.07 × 10^6^ kJ) (Fig. 3). However, we estimated that energy gain during 90 d of diurnal feeding in Nova Scotia was 2.89 × 10^6^ kJ for adult females and 3.53 × 10^6^ kJ for subadults and males. After accounting for costs associated with reproduction and maintenance costs during remigration intervals of varying durations (or 1 yr maintenance costs for subadults and males), we estimated that a single foraging season—including daytime foraging only—in Nova Scotia can account for approximately 51% of an adult female’s total energy needs in a breeding year, 29% of energy needs for an entire 2 yr remigration interval, and approximately 59% of annual energy needs for subadults and males (Fig. 3).

**Table 4:** Fattening rates for leatherback turtles based on allometric equation derived for migratory birds (ref.^49^) (i.e., “expected”), and based on actual calculations of net energy intake in this study (i.e., “estimated”). ^a^Ref.^19^; ^b^based on average 442 kg body mass.

% Mass increase^a^	Mass gain (kg)^b^	Expected rates		Estimated rates	
		Maximum fattening rate (kg d^−1^)	Days to reach foraging mass	Maximum fattening rate (kg d^−1^)	Days to reach foraging mass
26%	115		61		281
33%	147	1.879	78	0.410	360
40%	177		94		432

## Discussion

Because resources are typically distributed non-uniformly, places and times that offer predictable and abundant resources are disproportionately influential on animal ecology and population dynamics^35^. This is particularly true for migratory species, which must locate and exploit areas of high resource availability within their broad distributions to fuel energetic requirements of their life history demands^38^. Despite the cold water temperatures in this high-latitude foraging area in Atlantic Canada and the vast distance separating it from tropical breeding areas in the Wider Caribbean, leatherbacks from multiple breeding stocks migrate to—and show fidelity to—Canadian waters to take advantage of highly abundant, predictable prey resources^16–19,27,33^. Our analyses clearly demonstrate that this behaviour is energetically worthwhile: turtles can consume more than 200 kg, or more than 220 jellyfish per day (Table 2)—nearly 50% of their body mass daily—and these prey intake rates could fuel between 51% and 59% of leatherbacks’ total annual energy needs, and as much as 29% of a typical 2-yr reproductive cycle (Fig. 3). In fact, these energy intake rates are underestimates of the true energy intake by leatherbacks because we could not include potential nocturnal feeding rates for which no empirical data exist (see Methods). These results highlight the critical—and disproportionately high—value of continental shelf waters off Atlantic Canada for leatherbacks in the NWA. To fulfil the remainder of their energy requirements, leatherbacks access other foraging areas in the NWA^12,18,19,26,39^, likely timing their arrival in distinct areas to coincide with seasonal jellyfish ‘blooms’^18^. However, while there are several leatherback foraging areas in the broader NWA region, individual leatherbacks apparently show site fidelity, using specific foraging areas across years^27,28^. Energy intake calculations for other leatherback foraging areas^26,39^, other prey items^40^, and including potential nocturnal as well as diurnal foraging would produce more comprehensive energy budget estimates, thus improving our understanding of how the NWA leatherback population uses multiple foraging areas to meet their life history demands.

### Leatherback prey consumption rates and energy acquisition

The prey consumption rates that we quantified for daytime hours (>200 kg d^−1^ or ~50% of leatherback body mass daily) are comparable to previous daily estimates for leatherbacks based on calculations of energy acquisition rates required to meet reproductive and maintenance energy budgets (100 kg d^−1^ to 250 kg d^−1^, or 26% to 70% of total body mass daily)^41–43^. Direct observations of leatherback feeding rates (also limited to daytime hours) have been limited to two previous studies whose results vary widely^30,44^. Fossette *et al*.^44^ suggested that leatherbacks could meet basic daily energy demands by feeding on more than 14,000 small (4 g wet mass) jellyfish for ~4 h d^−1^, or approximately 59 kg d^−1^; these estimates were based on only 39 sec of video footage of two turtles in the Solomon Islands. In contrast, based on use of reported jellyfish wet mass^40^ (see Methods for details), Heaslip *et al*.^30^ reported that leatherbacks foraging in Nova Scotia may consume on average 260 large (~1 kg wet mass) jellyfish a day, or approximately 330 kg d^−1^. Likewise, our results demonstrated that leatherback energy consumption was more strongly influenced by prey size and biomass consumption rates than by prey capture rates. As noted in the Methods, our study updated—with an enhanced sample size—and re-analysed foraging data used by Heaslip *et al*.^30^, and discrepancies between results of the studies are due to methodological differences, such as how jellyfish sizes were estimated. Nonetheless, estimates of leatherback feeding rates—including ours, which are derived from the most robust dataset yet published—are based on brief time periods during daytime hours only because of logistical and technological limitations inherent in collecting video data from free-swimming turtles. Further, these observations are extrapolated to make inferences about consequences for bioenergetics and life history. Therefore, because the validity of these extrapolations is dependent on variation in availability (i.e., density and size) of gelatinous zooplankton prey, which can change considerably in space and time^44–46^, characterization of environmental drivers of leatherback prey availability should remain a research priority.

Our results also highlighted the importance of trade-offs between biomass intake rates—which are determined by prey distribution, not prey handling^31^—and energy costs incurred while actively swimming in cold waters in Atlantic Canada^41,43,47^ (Fig. 2). By targeting prey distributed close to the surface, in typically warmer water temperatures, leatherbacks can reduce the thermal gradient between body and water temperatures, thereby reducing thermoregulatory costs of maintaining high body temperatures^31,37,41,47^. However, even when thermoregulatory costs are high, leatherbacks can achieve net positive energy intake rates by increasing prey biomass intake—i.e., finding and exploiting larger patches of available prey (Fig. 2). Exploring how these factors vary at other leatherback foraging areas would likely demonstrate different energetic trade-offs and potential consequences for leatherback energy budgets.

### Leatherback energy intake compared to other taxa

Metabolisable energy intake—i.e., energy that is consumed and converted to meet energy demands—is constrained by trade-offs between prey availability (e.g., abundance, size, energy content) and consumer digestive capacity and efficiency^48–50^. The prodigious gelatinous prey biomass consumption rates documented for wild leatherbacks in this study and reported in previous observational and theoretical studies^30,31,42–44^, are necessary to compensate for the corresponding relatively low prey energy content. To illustrate this trade-off between high biomass consumption and low energy intake, we compared energy intake rates of leatherbacks to those of other migratory species (e.g., birds) and non-migratory species of body sizes similar to leatherbacks (e.g., large mammals) in two different ways. First, we calculated daily net energy intake and potential fattening rates using a mass-specific allometric equation derived for migratory birds^49^. We retained the same assumptions made in the original equation^49^ about energy conversion efficiency and energy content of fat. Second, we calculated daily, mass-specific, maximum net energy intake rates for leatherbacks (kJ kg^−1^ d^−1^) from our data and compared these values to those extracted or calculated from the literature for mammals and birds across several orders of magnitude of body sizes^48–53^. We restricted this exercise to published values of maximum energy intake, rather than simply maximum energy expenditure, which is more commonly reported for a wide range of species across taxonomic groups. These comparisons were meant to provide context for leatherbacks’ energy acquisition strategy, and were not intended to be exhaustive, phylogenetically controlled meta-analyses.

**Figure 4:**
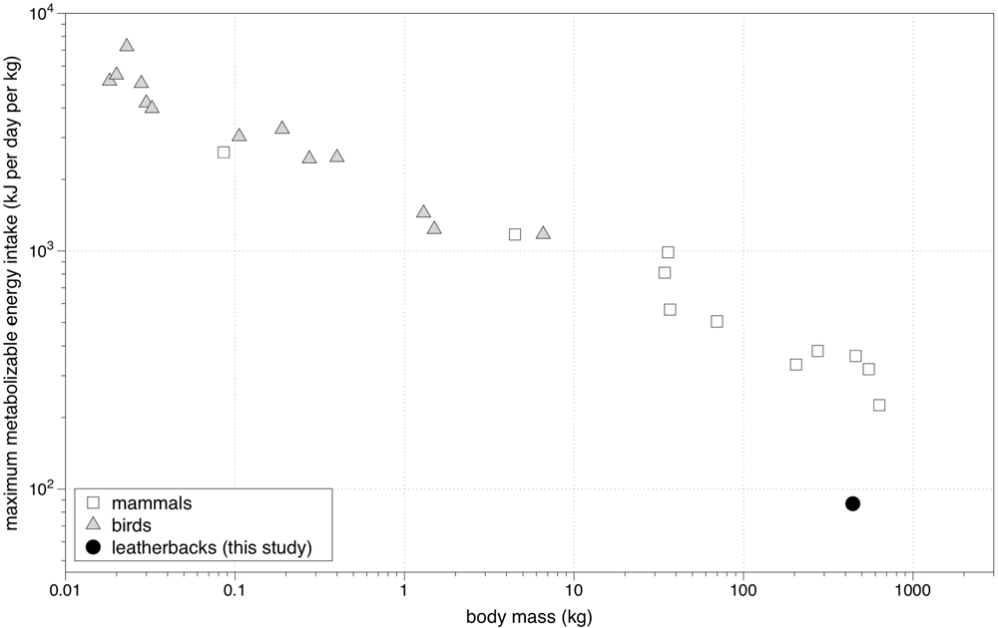
Maximum estimated daily metabolizable energy intake rates (i.e., kJ d^−1^ kg^−1^) for mammals (triangles), birds (squares), and leatherback turtles (filled circle). Data are restricted to values of maximum energy intake, rather than simply maximum energy expenditure, which is more commonly reported for a wide range of species across taxonomic groups. Data for mammals (species included: horse, cow, pig, dog, polar bear, Antarctic fur seal, Steller’s sea lion, weasel, rabbit, and human) and birds (species included: cackling geese, Landes goose, domestic fowl, black-bellied tree duck, lesser scaup, double-crested cormorant, kestrel, house martin, house sparrow, white-crowned sparrow, chaffinch, and thrush nightengales) from refs^48–54^; leatherback data from this study.

Results of these comparisons illustrated that leatherback energy intake rates are considerably lower than those expected for migratory and non-migratory species of similar body sizes (Table 4; Fig. 4). The maximum fat deposition rate that we calculated for leatherbacks (~0.4% body size d^−1^) was 30% lower than that predicted from the allometric equation^49^. That is, a ~400 kg leatherback must maintain an energy intake rate equivalent to that calculated in this study for at least one year (360 days) to achieve a ~33% increase in body mass (i.e., the difference in body mass observed between turtles in Canada and turtles of the same carapace lengths on breeding grounds^19^). In contrast, if the leatherback fat deposition rate was similar to that predicted from the original equation^49^, turtles could achieve this body mass increase in less than three months (~78 d) (Table 4). Similarly, leatherback energy intake rates—absolute (kJ d^−1^) and mass-specific (kJ^−1^ kg^−1^ d^−1^)—were 3.8 times lower than those estimated for other species of similar body sizes under high energy demands (e.g., lactation, high activity, etc.) (Fig. 4)^48–53^.

Leatherback metabolic rates are significantly different from mammalian and avian metabolic rates^42,54^, and the differences that we calculated here are probably related somewhat to taxonomic differences in metabolic physiology. Nonetheless, the degree of differences also reflects the low-energy prey resources on which leatherbacks rely compared to prey resources that fuel life histories of other migratory and non-migratory species. Therefore, these illustrative calculations highlight leatherbacks’ unique foraging strategy that must include efficiency both in energy conservation and in exploitation of typically ephemeral prey resources when they are highly abundant^55^. In this context, continental shelf waters off Nova Scotia are critically important to leatherback energy acquisition because they host large and reliable prey densities, which enable leatherbacks to forage continuously using short, shallow, energy-efficient dives in the photic zone at or above the main thermocline, and in warmer surface waters^27,31,47^.

### Consequences of variation in resource availability for leatherbacks

Decreased resource availability and/or quality could compromise the energetic profitability of a foraging area through higher incurred costs, lower energy intake, or both. In turn, this can have consequences for reproductive output, growth, and population dynamics^56^. For example, differences in availability of food resources (e.g., depth and, therefore, increased difficulty of access) and water temperatures differentially constrain energy intake rates in marine iguanas, which manifest in body size differences between allopatric populations in the Galápagos Islands^57^. Similarly, if leatherback prey were distributed at deeper depths associated with colder water temperatures, or if prey abundance were less predictably distributed across a broader area, turtles would have to expend more energy to find, capture, digest, and assimilate prey^36,43,44^. Such increased energetic expenditures by leatherbacks in foraging areas could have important consequences for overall energy budgets because net energy gain would be lower than in contexts similar to that documented in Atlantic Canada.

Comparisons among leatherback populations provide such an example of how variation in resource availability can influence life history. Foraging habitats used by leatherbacks in the NWA have significantly higher net primary productivity—i.e., a proxy for resource availability—than those used by leatherbacks in the East Pacific Ocean (EP), despite these foraging habitats being comparable in cumulative area^58^. Furthermore, leatherbacks in the EP generally perform deeper and longer dives that are presumably more energetically costly on average than do leatherbacks foraging in Atlantic Canada^31,59,60^. These differences in resource availability and foraging behaviour could underlie well-documented differences between estimated and observed energetic requirements and resulting variation in life history traits in these two subpopulations^31,43,61^. Specifically, despite lower estimated energetic costs, EP leatherbacks have longer remigration intervals (~4 yr) and lower fecundity (~65 eggs per clutch) than do NWA leatherbacks (~2–3 yr interval and ~80 eggs per clutch), indicating lower resource availability and higher energy expenditure for EP leatherbacks^43,56,58,61^. Consequently, stochastic resource availability has made the EP leatherback population less resilient than NWA leatherbacks to persistent anthropogenic threats^61,62^; EP leatherbacks have declined more than 90% in abundance in the past two decades, while NWA leatherbacks are far more abundant^20,25^. Fine-scale analyses of leatherback foraging behaviour and activity in the southeastern Pacific Ocean comparable to those conducted for leatherbacks in Atlantic Canada^30,31^ are not yet available. Such analyses would likely illustrate the relationship between divergent energy availability and resulting life history traits between these two sub-populations in greater detail. In particular, we hypothesize that regional variations in resource availability—i.e., NWA > EP—necessitate different foraging behaviours to acquire sufficient resources to meet life history demands, and the energetic consequences of these behavioural differences drive significant divergence in life history traits and population demography^31,43,58,61^.

## Conclusions

For migratory species whose distributions span broad geographic areas and habitats, reproductive success and survival can depend on efficiently locating and exploiting reliable, abundant resources to meet energy demands of their life history^1,3,5^. Our analyses demonstrate that the leatherback foraging area on the continental shelf of Atlantic Canada provides highly abundant prey resources that allow leatherbacks to meet a significant proportion of their annual or multi-annual energetic demands in a relatively short time period (Fig. 3), despite their focus on energy-poor prey (Fig. 4). The availability of such energetically valuable foraging areas allows NWA leatherbacks to grow to larger sizes and reproduce more frequently than their EP counterparts, which likely makes the NWA leatherback population more resilient to anthropogenic threats to its survival^62^. Therefore, Nova Scotia is disproportionately important to ensuring future stability and growth of the NWA leatherback population, making efforts to reduce spatially concentrated threats such as leatherback entanglement in buoy lines associated with fixed fishing gear in temperate shelf waters^19,26,62^ especially important. Furthermore, considering that NWA continental shelf waters are expected to warm significantly faster than warming rates projected for broader scales^63^, characterizing local and regional environmental drivers—and their sensitivity to future climate change—of productivity in the NWA^64^ should remain a research priority.

## Methods

### Field sampling and instrumentation

Fieldwork was conducted in temperate shelf waters off Neil’s Harbour, Cape Breton Island, Nova Scotia, Canada (approximately 47° N, 60° W) from mid-August to mid-September, 2008–2013.

Local and long-distance movements of leatherbacks tagged off Nova Scotia has been described using satellite telemetry^18,19,65,66^, but detailed information about energy acquisition patterns is lacking. Thus, we used a turtle-borne, integrated, continuous video-data logger incorporating suction cup attachment, remote release, GPS, and 1-sec temperature and depth sampling (Serrano-V tag, Xeos Technologies Inc., Dartmouth, NS, Canada). We deployed the video-data recorder by hand on 24 free-swimming foraging leatherbacks across five years (2008–2011, 2013). Data from four tags were excluded from analyses because deployments lasted < 1 hr, which was insufficient to adequately quantify diving and feeding behavioural data (though we did retain data from two turtles whose deployments lasted nearly 1 hr; Table 1). The video data provided a rare, nearly “turtle’s-eye” view of prey encounters and captures, and when coupled with dive data, facilitated highly detailed analyses of foraging behaviour and energetics.

Following the remote release of the video-data recorder, a subset of animals was successfully relocated and captured using a breakaway hoop net. Curved carapace length (CCL) and width (CCW) were then collected. We followed methods published previously^27,31^ using sexual dimorphism in tail length^17^ to assign sex to leatherbacks of ≥145 cm CCL only. In some cases, sex of adult females was confirmed by encounters on nesting beaches^27,33^.

Because not all turtles equipped with video-data loggers were subsequently captured, we were unable to record body sizes for the full sample of turtles in this study (range of CCLs: 143.5 to 164.0 cm, n = 9). For calculations requiring body size, we used an average of measurements made on turtles in this study (mean approximately 153 cm CCL). We estimated mass for individual turtles based on statistical relationship between CCL and body mass for the leatherbacks measured in Canadian waters between 2000–2013 (mass = [8.93*CCL]−925.35, r^2^ = 0.67, F_1,42_ = 85.1, p < 0.0001; ref.^17^ and unpublished data). In cases where we unable to measure CCL directly, we estimated mass based on the average CCL for turtles measured in this study.

### Bioenergetics modelling

We quantified energy intake and costs for individual turtles in this study to estimate the net energy gains by leatherbacks that forage in Atlantic Canada relative to their overall energy budgets. Below, we describe our approaches for estimating prey size and energy content; costs of thermoregulation and prey ingestion; and quantification of net energy intake relative to leatherback energy budgets. Recognizing that the Atlantic Canada leatherback foraging population includes subadult turtles, adult males that make round-trip migrations to breeding areas within a calendar year^18,19^, and adult females in different years of multi-year breeding cycles^32,66^, we calculated different energy budgets based on gender (adult males vs. females) and reproductive status (subadults vs. adults, non-reproductive vs. reproductive females).

#### Estimates of prey size

We quantified the number of prey encounters, captures, handling time and effort, and estimated sizes (average bell diameter) of jellyfish based on turtle-borne video footage during daytime hours only (Fig. 1). The camera provided a 90-degree field-of-view, so we were unable to count all jellyfish that were potentially visible to instrumented leatherbacks. For this reason, we almost certainly underestimate total prey encounters. Although Heaslip *et al*.^30^ reported bell measurements of *C*. *capillata* estimated from turtle-borne video, these were much smaller (11.2 ± 4.4 cm contracted bell diameters, range 3.1–22.7 cm) than specimens of *C*. *capillata* that were directly measured while their bells were expanded (30.3 ± 6.6 cm)^40^. Therefore, *C*. *capillata* measurements reported by Heaslip *et al*.^30^ were consistent with apparent versus actual bell diameters. Consequently, it was necessary to recalculate jellyfish sizes from apparent to actual sizes to refine estimates of leatherback energy acquisition based on the video records (see below).

Apparent sizes of objects–swimming jellyfish in this case–are underestimates of their actual sizes because they are perceived at some distance away from the point-of-view of the observer making the measurement. To account for this issue, we estimated sizes of jellyfish recorded in turtle-borne video using angular size and distance calculations. Briefly, we estimated the actual sizes of jellyfish bell diameters by measuring apparent diameters relative to the average head width of turtles in this study (~23 cm)^30^ immediately before turtles attempted to capture individual jellyfish (i.e., when jellyfish were positioned off the tip of a turtle’s nose). We noted the relative degree of jellyfish bell contraction or expansion at the point of measurement, and, where possible, made multiple measurements at different contraction phases to facilitate size conversions based on relative degree of contraction. We then estimated the distance from the tip of the turtle’s nose to the nuchal end of its carapace–which approximates the position of the video recorder lens–based on carapace length and using actual measurements of NWA leatherback head and neck lengths relative to curved carapace lengths (ref.^67^ and S. Fossette pers. comm.). We used standard calculations of angular distance to then convert apparent sizes of jellyfish that we measured directly from video footage to actual estimated sizes. In some cases, the turtle’s head was not in view continuously during the video (Table 1), likely because the camera had been attached slightly posteriorly and thus angled slightly upward. However, capture events could be detected, and many jellies could be measured. For jellies that were only partly visible, and thus could not be measured, we substituted the average jellyfish size for those that could be measured in a given video; the proportion of jellies that could be measured directly to those that were counted but not measured is referred to as the ‘measuring efficiency’ in Table 1. Thus, to measure jellyfish sizes while accounting for the position of the camera, the actual distance between camera and the turtle’s nose was increased in the calculations of actual (from apparent) jellyfish sizes.

#### Biomass and energy content of captured prey

We used the published relationship^40^ between *C*. *capillata* bell diameters and corresponding wet masses measured in the field to convert our estimates of *C*. *capillata* bell sizes described above to wet mass consumed during video recording sessions. This allowed us to calculate daytime biomass intake rates for all turtles. We then converted these estimated individual jellyfish masses to energy content per gram wet mass using published calorimetric measurements for *C*. *capillata* (0.2 kJ g^−1^)^40^. Note that Heaslip *et al*.^30^ also calculated energy content for *C*. *capillata*; however, those estimates appeared to have been based on an average bell diameter of *C*. *capillata* measured by Doyle *et al*.^40^ (~30.3 cm) and applied to all *C*. *capillata* consumed by individual turtles in video recordings (supplemental tables associated with^30^), rather than based on the contracted bell diameters measured and reported in the Heaslip *et al*. study^30^.

#### Energetic costs of thermoregulation and prey ingestion

We estimated energetic costs to leatherbacks of maintaining foraging activity and prey ingestion in cold water temperatures (*T*_*w*_) of Atlantic Canada. Leatherbacks maintain elevated body temperatures (*T*_*b*_) and a relatively high thermal gradient (*T*_*g*_ = *T*_*b*_ - *T*_*w*_) between ambient and core body temperature using a combination of anatomical and physiological adaptations and adjustments in swimming behaviour to generate and retain endogenous heat^41,68,69^. To estimate costs of thermoregulation, we generally followed a published approach^37,68^. Specifically, we used literature values and data obtained in the present study to estimate metabolic rates (MRs)—and thus energy expenditures—required to maintain high *T*_*b*_s and balance heat loss (*q*_*T*_) across body surfaces (shell, *q*_*S*_; flippers, *q*_*F*_) and heat transferred to ingested prey (*q*_*P*_) (in the present case, jellyfish temperature is assumed to be equal to *T*_*w*_), while accounting for energetic costs of specific dynamic action (SDA) associated with digestion of prey. Heat loss from the head and neck is assumed to be negligible due to significant peripheral insulation^70^. We assumed that blood flow continually redistributes endogenously-generated heat through the body core^71^, and that mean *T*_*g*_ reflects an internal thermal steady state^68^; this results in heat production—i.e., MRs—being equivalent to *q*_*T*_^37^.

We incorporated *T*_*w*_ data measured by the video-data recorder’s on-board thermistor to estimate the average water temperature experienced by each turtle. We then calculated *T*_*g*_ for each turtle using low, average, and high *T*_*b*_ measured for leatherbacks captured off Nova Scotia^37,47^.

First, we calculated heat loss across the shell (*q*_S_) following^68^ as:

1 $${q}_{{\rm{S}}}=({\rm{A}}\cdot k\cdot {T}_{g})\cdot {{\rm{L}}}^{-1}$$

where A is surface area of shell (m^2^), calculated as A = 0.049 mass^0.69^; *k* is thermal conductivity of shell, 0.25 W·m^−1^·K^−1^; *T*_g_ is the mean thermal gradient (°C); and *L* is insulation thickness, which we varied according to reproductive status, based on increased body mass at similar CCLs observed for NWA leatherbacks in Canada compared to when the same turtles are on nesting beaches^18^ (see below for input values).

We estimated heat loss across flipper surfaces (*q*_F_) as:

2 $${q}_{{\rm{F}}}=({q}_{S}/0.93)\,-\,{q}_{{\rm{S}}}$$

because *q*_F_ accounts for approximately 7% of total surface heat loss in cold water^68^.

We calculated heat transfer to prey (*q*_*P*_) as:

3 $${q}_{{\rm{P}}}={{\rm{M}}}_{{\rm{P}}}\cdot {{\rm{C}}}_{{\rm{P}}}\cdot {{\rm{T}}}_{{\rm{g}}}$$

where M_P_ is prey mass consumed (data from the current study, converted to kg·s^−1^); and C_P_ is specific heat capacity of prey (4186 J kg^−1^ K^−1^) (ref.^72^). Because we assumed that prey temperature is equivalent to *T*_*w*_, and warming of prey prior to ingestion is minimal^37^, calculated *T*_g_ is equivalent to the difference between core *T*_b_ and prey temperature.

We estimated rate of heat production due to SDA by applying a general equation for SDA in reptiles: SDA = 0.26 ME − 10.65, where ME is the meal energy (kJ) ingested per day based on published energy values for leatherback prey^40^ and prey consumption calculated for individual turtles in this study. The resulting value of SDA (kJ) for an average day’s foraging effort was then converted to W kg^−1^, using mass estimated for individual turtles based on the statistical relationship between CCL and body mass for leatherbacks described above.

#### Bioenergetics quantification

Finally, we integrated the energy gains and energy costs to calculate overall energy budgets for leatherbacks based on gender and reproductive state. Our goal was to estimate the proportion of leatherbacks’ overall energy requirements for breeding and non-breeding leatherbacks that can be acquired while foraging in Atlantic Canada. Thus, we only calculated energy intake while in Canadian waters, and did not consider energy intake at any other point during a migratory cycle. Obviously, leatherbacks must acquire resources in places outside of Atlantic Canada to meet overall energetic needs, but we made this simplifying assumption to explicitly quantify the relative importance of the well-defined, high latitude habitat in Atlantic Canada to wide-ranging NWA leatherbacks.

To calculate energy budgets of leatherbacks that exhibit seasonal foraging residency in Atlantic Canada, we generally followed published methods^43,44^. In particular, we estimated costs for foraging periods while in Canadian waters and combined these with energetic costs for “non-reproductive periods” for adult turtles (or “time away from Canada” for subadults) and “reproduction costs” for adult female turtles^43^. We first calculated energy budgets for time spent in Canadian waters, using 90 d as a typical foraging residency period length based on the temporal frequencies of leatherback sightings as well as tracking data in Nova Scotia during the past decade^27–29,65,73^.

Based on *in situ* measurements of leatherback *T*_*b*_ in Nova Scotia that indicated diurnal prey ingestion and nocturnal warming^36^, and because our empirical data on feeding and energy intake rates were restricted to daytime hours, we assumed that feeding – i.e., energy intake – only occurred during the day (~14 hr d^−1^; ref.^36^), and that energy expenditures during the night (~10 hr d^−1^) were equivalent to heat loss as calculated above. A few previous studies have inferred nocturnal feeding from documented ingestion events^74^ and apparent patterns of diel vertical migrations that suggested leatherbacks might be tracking prey that move from deeper water to waters near the surface from day to night^27,75^. However, Casey *et al*.^37^ showed that leatherback dive patterns in Nova Scotia did not differ between day and night, and concluded that nocturnal warming of leatherback body temperatures were indicative of endogenous heat production related to digestion of prey captured during the day. Our video-based observations of leatherback feeding (^31^, this study) and other studies^36,37^ have clearly demonstrated that leatherbacks forage almost exclusively in the photic zone, often within the top 20 m of the water column while in Nova Scotia. We acknowledge that nighttime foraging is possible, and by excluding estimates of nocturnal energy intake we are likely underestimating the energetic importance of Nova Scotia to leatherback energy budgets. However, available data on foraging leatherbacks—i.e., fine-scale dive behavior and feeding patterns, diel body temperature changes (diurnal cooling, nocturnal warming)—do not support the assertion of nighttime foraging in Nova Scotia. Furthermore, available data do not inform assumptions about how nocturnal feeding rates might vary in proportion to diurnal feeding rates observed directly^30,31,37^. For these reasons, we used a conservative assumption of exclusively daytime foraging in our energetics calculations.

We converted hourly biomass and energy intake rates estimated from daytime, turtle-borne video footage to daily rates to compute total energy intake during time spent in Atlantic Canada. Although previous analyses of leatherback movements in Atlantic Canada distinguished between areas of ‘transit’ (i.e., straight-line movements) and areas of ‘foraging’ (i.e., increased turning, slower speeds)^73^, recent analyses of fine-scale foraging behaviour of leatherbacks in this area demonstrated nearly continuous foraging, regardless of the trajectory of turtles’ movements through space^31^. Thus, we assumed that energy intake rates estimated from video data were samples of typical diurnal foraging periods, and could be applied to the entire residency period (~90d). We assumed that the insulation thickness layer, *L* (see equation [1] above) was 3.5 cm, which was an intermediate value based on measurements of leatherbacks that washed ashore dead in Canada^37^.

We then calculated energy costs for time away from Canadian waters. Although adult males migrate to and from breeding areas throughout the Wider Caribbean while subadults move south from Nova Scotia and remain at lower latitudes for the rest of the year^18^, we assumed that the time spent away from Canadian waters for both of these groups would be roughly the same cost per day based on the heat balance calculations described above. This assumption included a lack of feeding while away from Canada (see above). We used a *T*_*w*_ value of 24.0 °C as an average water temperature experienced by leatherbacks in lower latitudes during migration and breeding^19^. This *T*_*w*_ was used to calculate thermal gradients and thus potential heat losses and required metabolic rates. Because leatherbacks in Canada tend to be substantially more massive (~33%) than leatherbacks of the same carapace length measured on nesting beaches in the Wider Caribbean—a difference that is attributable to energy and mass gain between reproductive seasons^19^—we calculated a body mass for turtles away from Canada using a CCL-mass equation for turtles nesting in French Guiana (mass = [CCL ∗ 6.22] − 580.67; ref.^67^). Because this equation is based (necessarily) on data for nesting females only, using it to estimate body masses of adult males, subadults, and females in non-breeding years is a potential source of error. Likewise, we assumed that the insulation thickness layer, *L* (see equation [1] above) for turtles away from Canada was 2.0 cm, which was the low end of the range of shell thickness measurements of leatherbacks that washed ashore dead in Canada^37^. We then calculated the total “non-reproductive period” by subtracting the time spent in Canada (90d) and time spent near the breeding areas and/or nesting (60d) from remigration intervals (i.e., time between consecutive nesting seasons, RI) of 1 yr, 2 yr, and 4 yr based on average RIs (i.e., 2 to 3 yr) for NWA leatherbacks^17^.

For the Atlantic Canada foraging component for reproductive females, we used energy intake and expenditure calculations based only on video footage obtained from deployments on adult females. We used new data on energy gains obtained in this study to update the reproductive energy budget model developed originally by Wallace *et al*.^43^ and more recently by Wallace and Jones^76^. We estimated reproductive energy (RE) costs to adult females based on varying RIs, using the equation:

4 $${\rm{RE}}={\rm{N}}+{\rm{E}}+{\rm{I}}$$

where *N* = nesting activity, *E* = egg clutches, *I* = internesting periods. We used costs of *N* and *E* based on average clutch size of 79.7 eggs per clutch and average clutch frequency of 6 clutches per female^17,43,76^. To calculate costs of *I*, we used an average cumulative internesting period of 60d and estimated energetic costs using field metabolic rates measured for internesting leatherbacks^77^.

### Data availability statement

The datasets generated during and/or analysed during the current study are available from the corresponding author on reasonable request.

### Ethical approval and informed consent

Fieldwork in Canada was conducted in partnership with the Canadian Sea Turtle Network, and in accordance with guidelines of the Canadian Council on Animal Care, with review and approval by the Dalhousie University Animal Care Committee (permit numbers 08–077, 09–069 and 11–073), and Fisheries and Oceans Canada (license and permit numbers 2008–454, MAR-SA-2008–006, 323395, 323398, 326240 and 332697).
